# COMPARING THE ASSOCIATION BETWEEN DIABETES AND MORTALITY IN TWO INDEPENDENT BIRTH COHORT OF OLDER ADULTS IN MEXICO

**DOI:** 10.1093/geroni/igad104.1735

**Published:** 2023-12-21

**Authors:** Brian Downer, Jose Cabrero Castro

**Affiliations:** University of Texas Medical Branch, Galveston, Texas, United States; University of Texas Medical Branch, Galveston, Texas, United States

## Abstract

The prevalence of diabetes among middle-aged and older adults in Mexico has nearly doubled since the year 2000. This increase is primarily due to healthcare reforms that promoted diabetes screening. Insulin use and other treatments have also increased during the same period. Early diagnosis and treatment can reduce diabetes mortality risk. However, it is unclear if the increased diagnosis and treatment of diabetes in Mexico have reduced the mortality risk associated with diabetes in older adults. We used the Mexican Health and Aging Study to compare the association between diabetes and 5-year mortality in two independent cohorts of adults aged 60-69 in 2001 (1932-1941) and 2012 (1943-1952). The final sample included 3,694 participants in 2001 and 4,769 in 2012. Demographic variables included age, gender, education, living in an urban community, and having health insurance coverage. Self-reported health conditions included diabetes, hypertension, stroke, and respiratory disease. We used cox-proportional hazard regression models that included an interaction term for diabetes by cohort and adjusted for demographic and self-reported health characteristics. Overall, diabetes was associated with 2.56 times higher mortality (95% CI=2.18-3.00). The 2012 cohort did not have significantly different mortality risk compared to the 2001 cohort (HR=0.91, 95% CI=0.77-1.07). The association between diabetes and mortality for participants in the 2001 cohort (HR=2.52, 95% CI=1.95-3.26) was not significantly different from the 2012 cohort (HR=2.58, 95% CI=2.09-3.17). Our results indicate that diabetes continues to be a major risk factor for mortality in Mexico despite the increase in screening and treatment.

